# CLINICAL PHENOTYPING OF THE COGNITIVELY UNIMPAIRED IN THE AD SPECTRUM USING PET IMAGING

**DOI:** 10.1093/geroni/igae098.4338

**Published:** 2024-12-31

**Authors:** Cynthia Felix, Felix Joy Kollasserry, George Rebok, Beth Snitz, Pamela Ferreira, William Klunk, Suzanne Baker, Tharick Pascoal

**Affiliations:** University of Pittsburgh, Pittsburgh, Pennsylvania, United States; Indian Statistical Institute, Bangalore, Karnataka, India; Johns Hopkins University, Baltimore, Maryland, United States; University of Pittsburgh, Pittsburgh, Pennsylvania, United States; University of Pittsburgh, Pittsburgh, Pennsylvania, United States; University of Pittsburgh, Pittsburgh, Pennsylvania, United States; Lawrence Berkley National Lab, Berkley, California, United States; University of Pittsburgh, Pittsburgh, Pennsylvania, United States

## Abstract

Clinical phenotyping of cognitively unimpaired (CU) older adults who may progress to dementia is a pressing issue in Alzheimer’s disease (AD) since early interventions are the most effective. Out-patient cognitive screening commonly uses rapid and simple tests such as the Montreal Cognitive Assessment (MoCA) but its role in preclinical AD remains unclear. We hypothesize that sub-categorizing CU based on the presence of amyloid (A) and/or tau (T) pathologies, as measured by PET, can cognitively phenotype CU using MoCA. We studied 147 CU [CDR global score 0] older adults with MK6240 and Flortaucipir tau-PET and PiB or NAV4694 amyloid PET from the ongoing HEAD study. Based on tracer uptake, individuals were divided into A-T-, A-T+, A+T- and A-T- groups. They were evaluated using the UDS-3 battery. One-way ANOVA with Tukey’s correction for multiple comparisons, was used to test for significant differences between groups. With both MK6240 and Flortaucipir, MoCA total scores revealed significant differences between A+T+ and A-T- groups [mean: -1.74 (SE: 0.56), p: 0.013 and mean: -1.56 (SE: 0.6), p: 0.049] respectively. A+T- vs A+T+ was also significant with MoCA total and MoCA Delayed Recall using MK6240 [mean: -2.05 (SE: 0.77), p: 0.041 and mean: -1.15 (SE: 0.42), p: 0.033]. MK6240 shows more sensitivity than Flortaucipir. Therefore, MoCA, a simple and commonly used in-office neuropsychological test can detect cognitive dysfunction in Aβ and tau positive (A+/T+) CU older adults. This highlights the value of precisely characterizing preclinical AD subtypes that are more likely to develop AD dementia.

